# Modelling infectious viral diseases in swine populations: a state of the art

**DOI:** 10.1186/s40813-020-00160-4

**Published:** 2020-08-20

**Authors:** M. Andraud, N. Rose

**Affiliations:** Anses, French Agency for Food, Environmental and Occupational Health & Safety, Ploufragan-Plouzané-Niort Laboratory, Epidemiology, Health and Welfare research unit, F22440 Ploufragan, France

## Abstract

Mathematical modelling is nowadays a pivotal tool for infectious diseases studies, completing regular biological investigations. The rapid growth of computer technology allowed for development of computational tools to address biological issues that could not be unravelled in the past. The global understanding of viral disease dynamics requires to account for all interactions at all levels, from within-host to between-herd, to have all the keys for development of control measures. A literature review was performed to disentangle modelling frameworks according to their major objectives and methodologies. One hundred and seventeen articles published between 1994 and 2020 were found to meet our inclusion criteria, which were defined to target papers representative of studies dealing with models of viral infection dynamics in pigs. A first descriptive analysis, using bibliometric indexes, permitted to identify keywords strongly related to the study scopes. Modelling studies were focused on particular infectious agents, with a shared objective: to better understand the viral dynamics for appropriate control measure adaptation. In a second step, selected papers were analysed to disentangle the modelling structures according to the objectives of the studies. The system representation was highly dependent on the nature of the pathogens. Enzootic viruses, such as swine influenza or porcine reproductive and respiratory syndrome, were generally investigated at the herd scale to analyse the impact of husbandry practices and prophylactic measures on infection dynamics. Epizootic agents (classical swine fever, foot-and-mouth disease or African swine fever viruses) were mostly studied using spatio-temporal simulation tools, to investigate the efficiency of surveillance and control protocols, which are predetermined for regulated diseases. A huge effort was made on model parameterization through the development of specific studies and methodologies insuring the robustness of parameter values to feed simulation tools.

Integrative modelling frameworks, from within-host to spatio-temporal models, is clearly on the way. This would allow to capture the complexity of individual biological variabilities and to assess their consequences on the whole system at the population level. This would offer the opportunity to test and evaluate in silico the efficiency of possible control measures targeting specific epidemiological units, from hosts to herds, either individually or through their contact networks. Such decision support tools represent a strength for stakeholders to help mitigating infectious diseases dynamics and limiting economic consequences.

## Introduction

Infectious diseases represent a global threat to humans, livestock and wildlife animals and plants with potential cross-species transmission [1, 2]. Mitigating infection is therefore a one-health problematic, which needs to be addressed using all materials in hands, both in terms of research and resources. When referring to such problems, one commonly thinks about biological analysis, i.e. virological and immunological diagnostics, which are essential for the understanding of host-pathogen interactions [3–5]. Epidemiology aims at describing and analysing health disorders at the population level [6–8]. A specific branch of epidemiology often referred to synthetic epidemiology is dedicated to the identification of factors favouring or impairing infections of hosts, and how modifying these factors would alter the transmission process of the infectious agents. The last decades have seen the emergence of mathematical tools developed for the treatment of data related to infectious diseases dynamics [9–11]. Altogether, this toolset offers an analytic power to disentangle the interplay between virological, immunological and epidemiological processes through an integrative approach from within-host to population scales [12].

Swine production represents one third of meat consumption throughout the world, making pig industry one of the most important agricultural sector [13]. In this context, infectious diseases affecting pigs may therefore have dramatic consequences on the food chain supply. The recent outbreak of African swine fever (ASF) in China, the first pork meat consumer country, clearly illustrates the direct and indirect economic impact of the disease [14]. Indeed, a loss of more than 1 Million animals due to 165 declared ASF outbreaks was recorded throughout the country [15], inducing a global shift in world pork market with huge economic consequences. Apart from ASF, swine industry is also affected by several enzootic viral diseases, most of which impair the productivity of herds, being therefore of huge economic importance (e.g. Porcine Reproductive and Respiratory syndrome (PRRS), Porcine Circovirus of type 2 (PCV-2)), and some of which being of public-health importance due to their zoonotic nature, such as swine influenza virus (SIV) and hepatitis E virus (HEV). In both cases, in-depth understanding of infectious diseases dynamics is needed to adopt appropriate solutions to mitigate the infectious pressure among the host population.

The present paper will review the use of mathematical dynamic modelling addressing viral infectious diseases in pigs. The aim was to assess how the field is represented in the literature and to characterize which kind of modelling choices and approaches are made according to the objectives.

## Methods

A literature search on PubMed and Scopus databases was performed on March 28, 2020, including articles with the following terms as keywords, title or abstract terms: (*pig* OR *swine*) AND (*virus* OR “*viral infection”* OR “*viral disease”*) AND (*simulation* OR *mathematical* OR *stochastic* OR *estimation* OR *inference*) AND (*model* OR *modeling* OR *modelling*). In the present review, being dedicated to viral infectious diseases modelling, we decided to swipe relatively large, to include studies presenting mechanistic formulation of viral spread at different scales. This led to the preselection of 907 records (Pubmed: 458; Scopus: 449) from which 273 were duplicates.

**Fig. 1:**
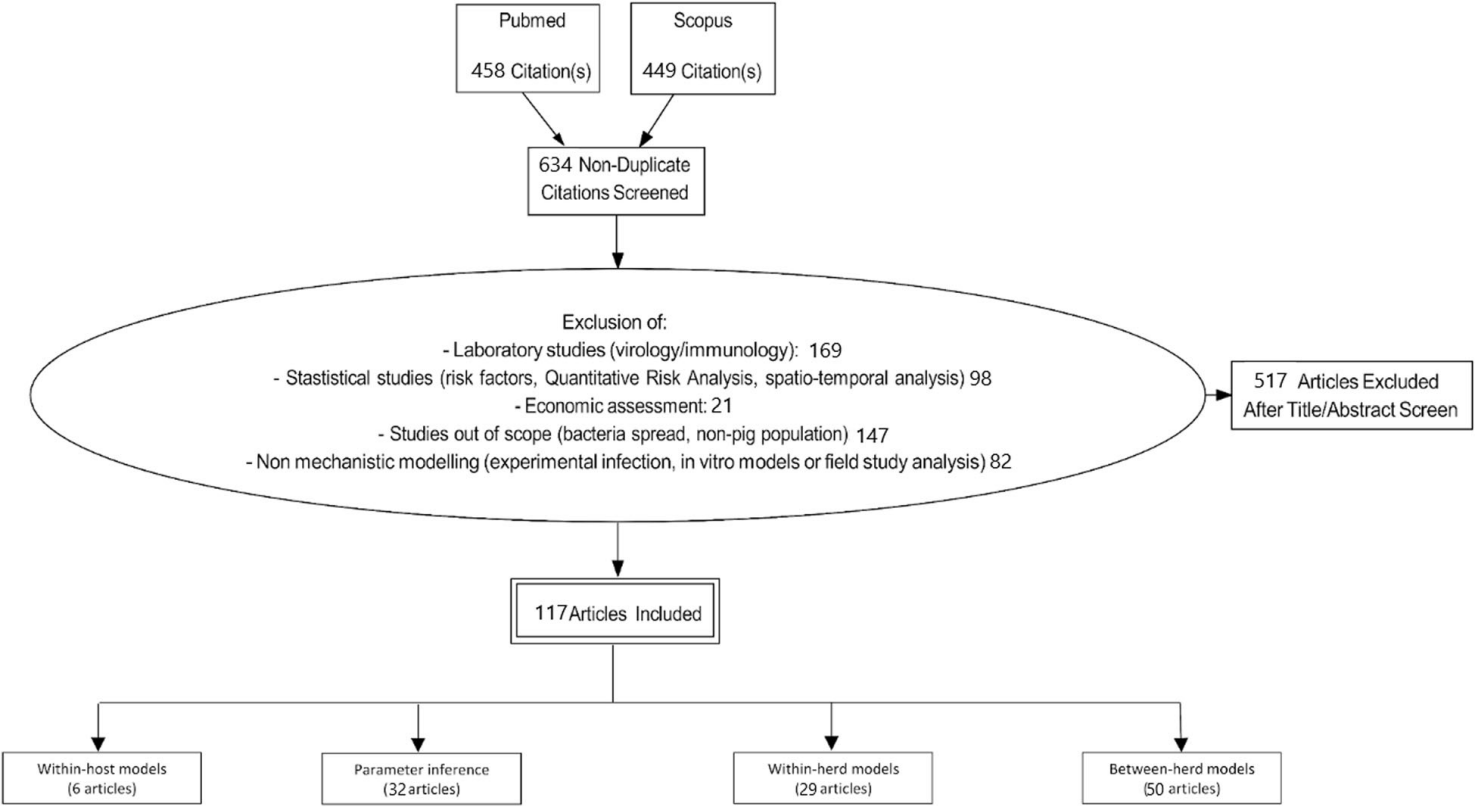
Prisma flow chart of the selection process

The titles and abstracts of 634 articles published between 1994 and 2020 were screened to select articles presenting dynamic models of viral diseases in domestic pig production system. All mechanistic modelling approaches representing the transmission of pathogens in swine populations were first selected. Therefore, papers on virological or immunological aspects were excluded (169 papers), as well as statistical studies for risk factors analysis, quantitative risk analysis (98 papers) or models aiming at economic evaluation of interventions in infected premises (21 papers). Finally, 147 studies focusing on bacteria or alternative host species were discarded as well as 82 studies not considering mathematical approaches (e.g. experimental infection, in vitro models or field study analysis). Prisma flow diagram is displayed in Fig. 1. A bibliographic analysis was first performed to have a global overview of our selection using bibliometric package from R software [16]. This analysis allowed for disentangling the studies in regards with the modelling frameworks and objectives.

With these considerations, selected records were split into four different categories owing to the objectives of the studies:

(i) Understanding host-pathogen interaction (6 studies),
(ii) Inferring epidemiological parameters (32 studies),
(iii) Assessing infection dynamics and mitigation measures at herd scale (29 studies),
(iv) Assessing surveillance and control measures at large geographical scale (50 studies).

For each of these themes, modelling strategies and objectives will be generally discussed with some concrete references derived from the literature review.

## Results

### Descriptive analysis

**Fig. 2:**
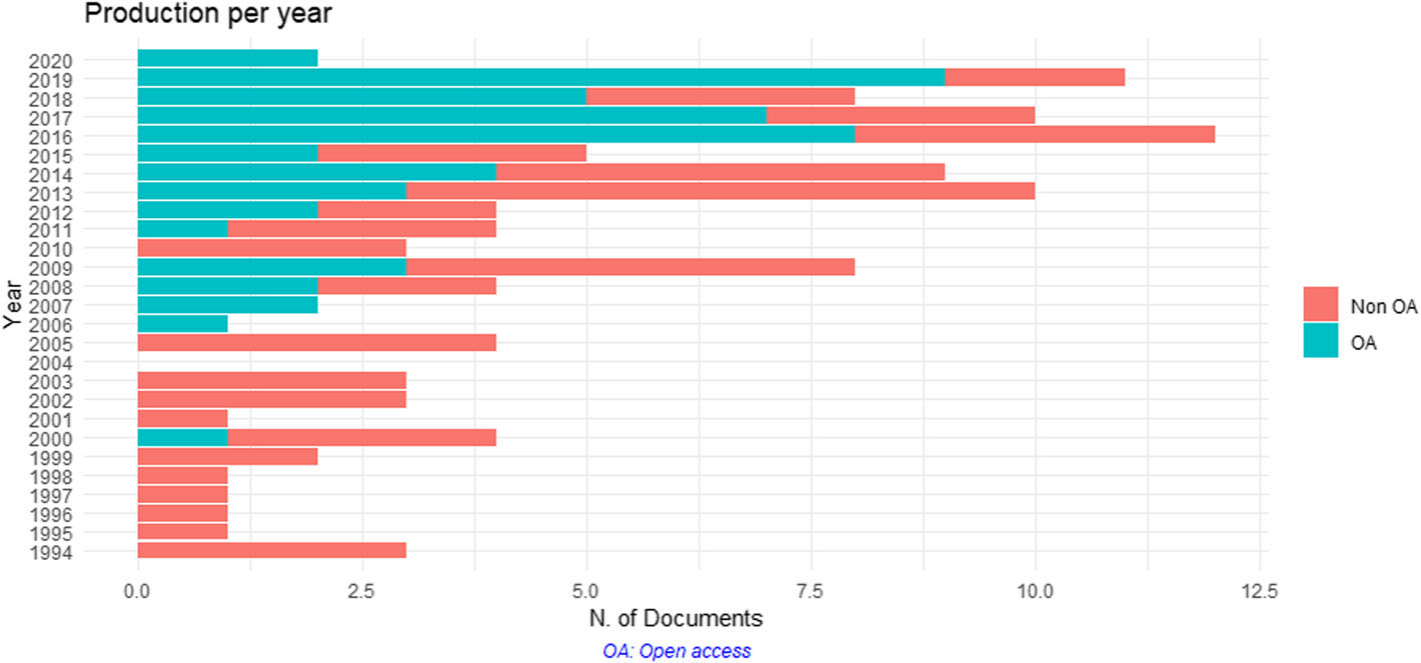
Evolution of scientific production in pig disease modelling per year

Based on the selection process, 117 records finally met the inclusion criteria (Supplementary Material 1). The earliest records were published in 1994, and these modelling studies were dedicated to pseudorabies virus, responsible of Aujeszky’s disease, in the Netherlands and United-States, respectively [17, 18]. As shown in Fig. 2, reflecting the evolution of the number of studies per year, the rise of modelling studies on viral diseases in pigs occurred in the mid-2000’s, with a prominence of open access publications in the last 4 years. Veterinary journals have been mainly targeted (representing about 65% of publications) but five journals with broader scopes are in the 10 major journal list (Supplementary Material 2).

**Fig. 3:**
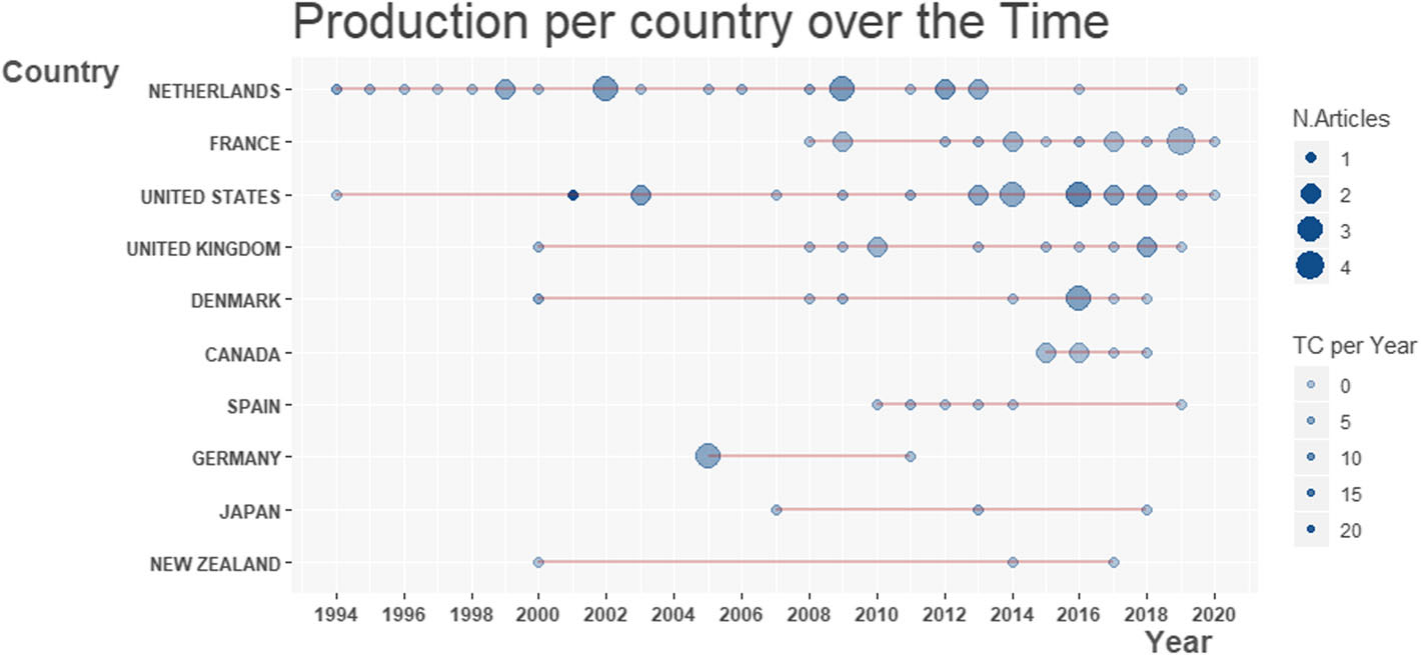
Evolution of scientific production per country in pig viral infection modelling per year (N.Articles: Number of published Articles; TC per Year: Total Citation count per year)

This specific research field was mostly treated by European teams, especially Dutch, French and UK research centers (with 25, 17 and 12 first-author publications, respectively), along with US teams accounting for 17 publications (Fig. 3).

**Fig. 4:**
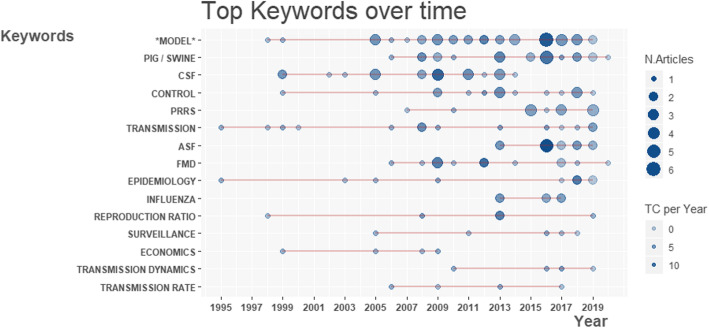
Keyword occurrences in time (N.Articles: Number of published Articles; TC per Year: Total Citation count per year). Terms including “Model” were gathered as well as keywords “Pig” and “Swine”

The frequency of keyword use over time is represented in Fig. 4. Queried keywords were systematically retrieved in the selected records, several complementary ones were also identified. Apart from search terms, viral infections of major economic importance were on the top of authors’keywords along with terms related to modelling objectives. The major viral diseases affecting pigs being main concerns for the swine industry are often used as primary keywords. Classical swine fever (CSF) was on the top of keywords with a total of 21 occurrences from 1999 [19, 20] to 2014 [21]. PRRS, ASF, FMD, SIV and HEV figured out in the 15 most used keywords, reflecting the variety of pathogens targeting pigs. CSF, FMD or PRRS have been involved in modelling studies continuously since 1994, while SIV and ASF have been only investigated recently, after the 2009 swine influenza pandemic and the emergence and spread of ASF in Europe respectively. The applied nature of modelling studies in swine populations is also highlighted by specific keywords corresponding to the main objectives of the studies, control of pathogen being the common purpose of all studies (Fig. 4). However, control strategies may take different forms depending on the disease under consideration. Indeed, two groups of infectious agents can be established corresponding to epizootic and enzootic diseases and raising different issues to modellers. Indeed, enzootic diseases, such as PRRS or SIV, which cause huge economic losses for swine producers, raise the question of their persistence on farms, despite vaccination programs. Here, the question of within-farm infection dynamics is of primary importance along with the evaluation of alternative, or complementary mitigation strategies, such as modifications of husbandry practices. In contrast, the control of epizootic diseases such as CSF, ASF or FMD, requires evaluating the between-herd spread, as well as surveillance strategies to evaluate the capacity and the cost-effectiveness of the different control measures. Modelling control of infectious diseases requires an in depth understanding of the transmission process at all scales, through the specification of mechanistic relationships, the identification and the estimation of key parameters, as the basic reproduction number, to feed simulation tools.

### Comprehensive review of modelling approaches and objectives

#### Understanding host-pathogen interaction

The analysis of infection characteristics at the host-scale may provide important insights on the infection dynamics at population scale. Indeed, the response to infection may be highly heterogeneous between hosts impacting the disease dynamics at population scale. The simplest models considered the interaction at the cellular scale with the infection of target cells and viral replication and release, and has been used to evaluate the impact of the FMD infectious dose on within-host viremia kinetics [22]. The other five studies were dedicated to within-host kinetics of PRRS virus infection, which induces an immuno-modulation, potentially favouring co-infections, and is therefore of particular importance having regard to host-pathogen interactions. Doeschl-Wilson and Galina-Pantoja [23] presented models of increasing complexity to account specifically for host immunity. This work allowed building up an integrative approach to represent the interaction between viral replication and the immune response, to further understand the persistence of viremia and between-host variability of PRRS [24–26].

#### Inferring transmission parameters (blue highlight in Supp. Mat. 1)

Mathematical formalisation of biological systems relies on the identification of state variables and mechanisms governing between-state transitions. Parameter values play an essential role to feed simulation tools. One may distinguish two frameworks for parameter estimation. First, the inference from experimental data: experimental studies offer the opportunity to analyse in a fully controlled environments specific characteristics of viral transmission. Different strategies exist for parameters estimation, all relying on relevant data gathered on the field or experimentally and on pre-established model structures [27]. Such approach requires a clear identification of knowledge gaps and the development of specific experimental designs to obtain accurate data to fill these gaps [28]. To estimate epidemiological parameters, data usually consist in the follow-up of infection sequence of naïve pigs in contact with inoculated ones [29–31]. Twenty-three articles from our selection focused on the analysis of experimental data. Such analyses were initiated by De Jong, Bouma and Kimman, with a particular focus on pseudorabies virus, the transmission of pathogens being quantified in different contexts depending on the contact structure between individuals or the immune status of the host [17, 32, 33]. The methodology was then adapted to different viruses such as PCV-2, FMD or more recently ASF, for which direct contact with penmates and indirect contact between animals housed in neighbouring pens are the main transmission routes [28, 34, 35]. The inference framework was also extended to account for alternative transmission routes, using specific data related to the viral load present in the air for SIV or in the environment (HEV) [29, 36]. For all these approaches on parameter inference in an experimental context, the underlying model is a stochastic Susceptible-Infectious-Recovered model where the infection pressure – or force of infection (FoI) – exerted on naïve animals is proportional to the prevalence of infected pigs in the population. The proportionality factor, denoted *β*, represents the transmission rate, i.e. the average number of new cases produced by one infectious animal per time unit [9]. This parameter, along with the duration of the infectious period, are the key components for the evaluation of the basic reproduction number (*R*_0_). Parameter estimations are usually based on likelihood approaches: the parameter space is scanned to find the optimal values that maximize the probability of observing the whole dataset given the model structure. Deterministic approaches, using minimization algorithms, were first used, but Bayesian frameworks are more and more present in the literature to unravel unobserved processes [37–39].

These Bayesian approaches are also used for parameter estimation from observational data [37, 40]. The rise of approximate Bayesian computation methods provides new toolsets for parameter inference [41]. This method consists in the definition of a metric to measure the adequacy of the model outcomes with observed data. Simulations are then performed with parameter set drawn from prior distributions. The parameter set are accepted and stored if the model outcomes are close enough to the actual data, based on the predefined metric. The selected parameter values define the posterior distribution for the parameter set. Guinat et al. [42] used this method to estimate epidemiological parameters from ASF mortality data in nine pig farms in Russian Federation. With *R*_0_ ranging from 4.4 to 17.3, this study highlighted farm specific spreading patterns, which were likely related to husbandry and biosecurity farm specific measures.

Parameters are essential components for model development and analysis. Their roles and values may modify in a critical way the behaviour of the outcome variables. Insuring the robustness of parameter values allows for stronger analysis of external factors influencing the infection dynamics at population scale.

#### Assessing infection dynamics and mitigation measures at herd scale (yellow highlight in Supp. Mat. 1)

Twenty-nine records were dedicated to dynamic models of viral infection transmission at the herd level with a similar goal: representing the infectious process in regards with epidemiological knowledge to test strategies for mitigating the viral spread [43–45]. These models particularly focused on enzootic pathogens, i.e. PCV2, SIV, PRRS gathering 17 articles. Population models capturing the demographic processes in swine herds are therefore coupled with specific epidemiological models representing the infection history for the pathogen under study. For pigs reared in groups of relatively small sizes, stochastic frameworks have been privileged [43, 46–49], although deterministic approaches based on ordinary differential equations were also developed. The latter were either theoretical models with in depth mathematical analysis, providing insight into the model behaviour according to epidemiological assumptions or parameter values [50–53], or applied models with an accurate representation of the farm systems [54–56]. For illustrative purpose, the issue of on-farm persistence of SIV represent a quarter of the selected articles. The set of companion papers by Reynolds et al. [56] and Etbaigha et al. [54] dealt with the spread of influenza virus in breeding and farrow-to-finish pig herds using deterministic modelling frameworks. The herd structure was described in details accounting for animal movements between different barns. The authors showed how easily the virus became endemic once introduced in a free herd. Indeed the frequency of piglet birth induced a regular inflow of susceptible individuals allowing for viral persistence. Only homologous vaccination – assuming total vaccine-induced protection – strategy was able to mitigate the infection. However, such level of protection is entirely theoretical and several factors may impair the development of the immune response. (e.g. maternally derived antibodies (MDA)). Stochastic models were also developed to address the influenza issue. Pitzer et al. [57] represented three types of herds, i.e. farrow-to-finish, wean-to-finish, and finisher farms. The herds were represented with barns housing pigs of similar physiological statuses and assuming specific contact rates between these barns to assess the within-herd persistence of SIV according to herd size. A critical herd size (CHS) of 3000 individuals was evidenced for SIV persistence with assumed *R*_0_ values between 1.5 and 2.5. However, experimental studies revealed much more effective transmission with *R*_0_ values ranging from 10.4 to 14.8 which would lower the estimated CHS. Cador et al. [58, 59] identified MDA as playing a major role in the persistence of SIV on farm. A stochastic model was developed considering batches as main epidemiological units, accounting specifically for the relationship between the sows and their litters through delivery of maternally derived immunity. The partial protection conferred by this passively acquired immunity was found to slow down the infectious process in young piglets but extended the infectious process at the batch level, favouring between batch transmission. This modelling framework was further extended to represent the spread of two antigenically distant SIV subtypes. Throughout the simulation process, concomitant infections by the two subtypes represented 16% of infection events, potentially inducing a risk of reassortment. The evaluation of different control measures showed that vaccination poorly managed to reduce SIV transmission, confirming previous results from other studies.

The study of some pathogens may require considering thinner epidemiological scale with the use of agent-based models. Such approach allows for the inclusion of individual variability, which may play a role in the infectious process at the population level. This was especially the case for HEV due to the evidence of transmission enhancement in pigs co-infected with immune-modulating viruses (IMV; e.g. PRRS). Salines et al. [60] analyzed the spread of HEV in farrow-to-finish pig farms through the development of an agent-based framework describing the co-circulation of HEV along with an immune-modulating virus. Such approach allowed for the analysis of modifications of husbandry practices acting on individual animals, but having a more general impact on the course of infection (e.g. cross-fostering, pig mingling in pens) [43]. A higher HEV persistence probability was evidenced in the presence of IMV, as well as in herds managed with intensive batch-rearing systems (20 batches) for which the birth of susceptible individuals is more frequent. The herd structure and husbandry practices, such as cross-fostering and piglet mingling, were also highlighted as important factors for HEV management.

Within-herd models are also developed in view of surveillance evaluation purposes, especially for notifiable diseases such as CSF or ASF. Backer et al. [47] evaluated the effectiveness of mortality-based detection measures of CSF applied in Netherlands in 2010 and found that it was strongly dependent on the physiological states of the animals. The high natural mortality rate (apart from CSF) in piglets impaired the detection process, as was the case in breeding animals due to low CSF-associated mortality. The protocol was nevertheless effective in fattening units where CSF-mortality could be disentangled from natural death, shortening the detection delay from 2 days. *Costard* et al. [61] integrated data on farmer’s behaviours to a within-herd ASF transmission model to assess their risk on virus spread due to release of infected animals. This study highlighted the importance of backyard and small-size herds and suggested those as potential target for surveillance purpose since they may deliver infected animals through their trade contact network.

#### Assessing surveillance and control measures at large geographical scale (Orange highlight in Supp. Mat. 1)

Based on the selection process, between-herd infection spread was assessed in 50 research studies, along with evaluation of surveillance and control measures. The earliest model focused on pseudorabies transmission between herds, showing the importance of Aujeszky disease in the 90’s and the need for modelling studies to evaluate the feasibility and efficacy of strategies for its eradication in several European countries [18]. Buijtels et al. [62] built a model considering the herd as the epidemiological unit and used a transition matrix including 24 heath states to analyse the impact of vaccination strategies. Van Nes et al. [63] developed a model considering both within- and between-herds transmission, in order to analyse the impact of control measures at farm-level and the infectious dynamics at larger scale.

In early 2000’s, a particular focus was made on foot-and-mouth disease due to a large outbreak in the UK. Fifteen studies focused on FMD considered multispecies models including cattle, sheep and pig herds. As such, pigs were considered in these models as potential hosts for FMD infection, but were not the target of the studies. Although pigs played a minor role in 2001 UK epidemics [64, 65], the role of swine holding was expected to be much higher in Denmark or Netherlands, clearly extending the outbreak persistence [65, 66]. These studies first aimed at unravelling the roles of the different transmission routes on between-farm transmission, such as animal movements or local spread through airborne route [67–69]. Control measures, consisting in ring-vaccination or ring-culling, could then be implemented in the models to evaluate their efficiency on the final size of the epidemics and the outbreak duration. Large radius ring-vaccination (> 10 km round from infected herds) was found to be relatively efficient to limit the outbreak size compared with ring-culling strategies [69–71].

About 15% of the between-herd transmission models focused on CSF (18 papers), a regulated disease, with similar objectives as the ones applied to FMD. Modelling frameworks could be shared for the two problematics [68, 72]. However, conversely to FMD, CSF is specific to swine, and multi-scale modelling was therefore more frequent in this case, representing explicitly the on-farm dynamics of infection [21, 73, 74]. Backer et al. [75] developed such a framework combining the within-herd transmission, considering mechanistic transmission between penmates and neighbouring pens, and the between-herd transmission, using distance kernel transmission from neighbouring herds. This framework allowed the evaluation of control strategies consisting in ring-culling or ring-vaccination from 1 to 5 km radius. Two km-ring vaccination was evaluated as efficient as ring-culling. However, vaccination would induce a higher number of undetected epidemics, and a specific end-screening protocol, targeting vaccinated herds, would be necessary. The authors further studied the impact of additional administration of antivirals in sows [76]. This treatment was found to improve significantly the control measures previously tested, showing the importance of the within-herd process representation, including breeding and growing pigs, to go deeper in the representation of detailed control strategies.

Generic modelling frameworks were also developed in view of homogenization and reproducibility of the methodology used for between-herd models. Four major frameworks were used to model pig viral infections at large geographic scales, namely: InterSpread [ISP] [77–79], North America Animal Diseases Simulation Model [NAADSM] [80–83], Danish Technical University - Davis Animal Disease Model [DTU-DADS] [84, 85], and Between-Farm-Animal Spatial Transmission [Be-FAST] [21, 86, 87]. All models are fed with herd characteristics (farm types, sizes and locations), and account for direct, indirect, airborne and environmental transmission routes. Boklund et al. used ISP framework to evaluated nine control strategies towards CSF in Denmark based on their effectiveness in reducing disease burden and infection durations. The NAADSM framework was used to evaluate the consequences of the introduction of a highly virulent strain of Porcine Epidemic Diarrhoea (PED) virus in a densely populated area in France [82]. The high responsiveness when the first detection occurs, with movement restriction and stamping out of infected herds within 10 days, appeared essential for limiting the infection spread. This framework was also used to assess the spread conditions of PRRS virus in Canada, highlighting the role of indirect contacts through truck sharing, and thus the importance of strict biosecurity measures for mitigating the transmission process [83]. Be-FAST and DTU-DADS model account explicitly for within-herd spread modules, assuming homogeneous mixing, to assess the overall infectious pressure exerted on susceptible herds, through local transmission (e.g. airborne) or trade exchanges. They also integrate an economic module to evaluate the cost-effectiveness of intervention strategies. Recently Halasa et al. published a set of papers describing the consequences of introduction of ASF in Denmark and assessed the efficacy of control measures using the DTU-DADS modelling framework [85, 88, 89]. The Be-FAST framework was used to study the transmission patterns of ASF in Sardinia, the only European region where the virus became endemic after its introduction in 1978 [87]. The role of unregistered domestic pigs and wild boars was clearly highlighted as a key component for ASF persistence on the island.

## Discussion

The present paper provides an overview of mathematical models applied to viral infections in pig populations. With a broad search algorithm, more than 100 publication records meeting the selection criteria were identified. The analysis of authors’ keywords revealed common scopes: identification of key parameters, evaluation and control of disease spread at herd scales, and modelling spread pattern at large scale to evaluate control measures for regulated diseases.

Swine production is an organized system from within farm, where pigs are housed according to strict predefined husbandry practices (all-in-all-out flow of animals, batches of animals of similar physiological statuses, prophylaxis measures), to commercial trade between production sites. These characteristics lead to different contact structures depending on the scale we’re actually interested in, which were already identified as potential weakness points in regards with pathogen spread and persistence [27, 43, 90–92].

Understanding viral infection dynamics in pig herds has been mainly based on observational and experimental studies describing real phenomenons in the field or evaluating assumptions in a controlled way in experimental settings. If several bias and confoundings can be encountered in observational studies, extrapolation of experimental findings to the real life is often challenging. Modelling studies can overcome those drawbacks by putting together all the information coming from different sources in a comprehensive way to represent mechanistically the biological events.

Within-host models are broadly used for human infectious agents, such as human immunodeficiency virus or influenza [93–95], but mathematical modelling of the host-pathogen interactions represents only six records in our selection of modelling studies dealing with pig diseases. Recent studies on SIV and PRSS revealed an impairment of the immune response when challenging piglets having maternally derived antibodies [96, 97]. These impairments were shown to be a key factor favouring the persistence of SIV on farm [59] and an additional source of host heterogeneity in regard to PRRS virus [96]. The kinetics of MDA, and related neutralizing antibodies, could therefore be a key component to understand the vaccination efficacy in the field. Immuno-epidemiological models, combining within-host immunological processes with epidemiological models at population scale, could help optimizing vaccination strategies.

The role of within-herd structure was broadly studied for enzootic agents [43, 56, 98], merging different within- and between-herds infection dynamics frameworks, have been used in the last decade (e.g. NAADSM, InterSpread, DTU-DADS or Be-FAST), mostly to study the spread of regulated diseases. A homogeneous mixing assumption is generally used to represent within-herd infection dynamics coupled with between-herd transmission module [43, 56, 98], but Kinsley et al. showed that the incorporation of accurate population dynamics in herds might shed new light on the actual infectious process [99]. A recent study on HEV spread integrated the compartment structure in pig herds in the SimInf framework [100], a modelling framework originally developed to study VTEC-O157 spread among cattle herds in Sweden [101]. Although more complex; this model accounted for realistic representation of the population demographics, together with the commercial network between production sites based on batch-rearing system. These examples illustrate the need of coupling models across scales. All scales are strongly related and acting on one link in the chain can modify the whole system, with beneficial or detrimental effects.

Halasa et al. [102] compared the outcomes of the Interspread and DTU-DADS frameworks using FMD application for reference disease, showing slight differences of spread patterns and control policies efficiency. Roche et al. [103] performed similar work, comparing modelling frameworks from five countries, namely Australia, New Zealand, USA, UK and the Netherlands. Although the results varied quantitatively, the main conclusions on the effectiveness of control strategy were robust for all models, which clearly help to be confident with their conclusions. In-depth analysis of models’ outcomes, based on similar assumptions, could certainly help understanding the impact of the model structures in terms of predictions.

There is a need at different levels and to different aims of modelling based information on viral infectious disease in pigs. The field of application and the representation structure is somehow correlated with the specific target/final user. Hence, models on regulated diseases will be often developed at the between herd scale and will address questions from policy makers in animal health [68, 74, 104, 105], whereas models developed for enzootic diseases are more generally developed on a herd level scale to answer more applied questions on disease control rather relevant for veterinarians and farmers [43, 57, 60]. These models can put together available data from observational and experimental origin and can provide estimates of non measurable facts. A wide range of methodologies have been developed to this aim, either based on representation of field data by a dynamic model or by using a simple model describing the infection dynamics occurring in a controlled experimental setting [29, 35, 42]. These developments have been used for transmission parameter estimation per se, but also to evaluate in experimental conditions the impact of a control measure (often vaccination) on virus transmission [106, 107]. These approaches based on data-designed models need to be distinguished from pure model development of complex structured population in which a pathogen or a combination of pathogens is spreading. This latter strategy has been often developed in porcine health management to understand infection dynamics in a comprehensive way by filling the gap of unobserved phenomenon (by observational or experimental studies) [48, 49, 60]. Hence the objective is rarely the pure prediction per se with the exception in specific cases of regulated diseases for the question of the impact of the pathogen in case of introduction in a free territory. Those models are rather used to evaluate assumptions based on in-silico experiments where a tested scenario is evaluated towards a control one. The magnitude of the effect is therefore rather expressed relatively to a baseline than an absolute estimate of the impact, but is extremely efficient in evaluating ex ante the impact of control strategies on an epidemics at different scales and potentially incorporate an economical assessment [62, 78, 85]. The advantage of modelling is the quasi absence of limits in the diversity and numbers of combinations of intervention strategies to be evaluated, which would generally be not possible to assess in real life.

Integrative modelling from within-host to between-herds virus dynamics is clearly on the way, but model coupling remains challenging [12, 108]. The rise of computer technology allowed for development of computational tools to address biological issues that could not be unravelled in the past, providing decision-support system to stakeholders. The emergence of artificial intelligence in the field of epidemiology may be a key for unifying multiple paradigms into a single multiscale framework [109]. The understanding of how control measures applied at one scale impact the system at up- and downward scales is essential to have a global overview of their efficiencies. The era of big data offers tremendous quantity of information on diseases spread throughout the world, requiring the development of specific methods for their analysis. One may easily understand the cooperative game that is currently played, gathering interdisciplinary research towards the same goal: a better understanding of infectious diseases dynamics for efficient control policies.

## Supplementary information

**Additional file 1: Supplementary Material 1.** Bibliographic Detail on records meeting the inclusion criteria: 117 articles according to their modelling structure (white: Within-host models, blue: parameter estimation, yellow: within-host model, orange: between-host model).

**Additional file 2: Supplementary Material 2.** Top journal list for modelling studies on infectious diseases dynamics in pigs.

## Data Availability

Not Applicable.
