# Supplementary figures and images for: Modelling infectious viral diseases in swine populations: a state of the art
Source: Porcine Health Manag. 2020 Aug 20;6:22. doi: 10.1186/s40813-020-00160-4 (PMC7439688; doi:10.1186/s40813-020-00160-4)

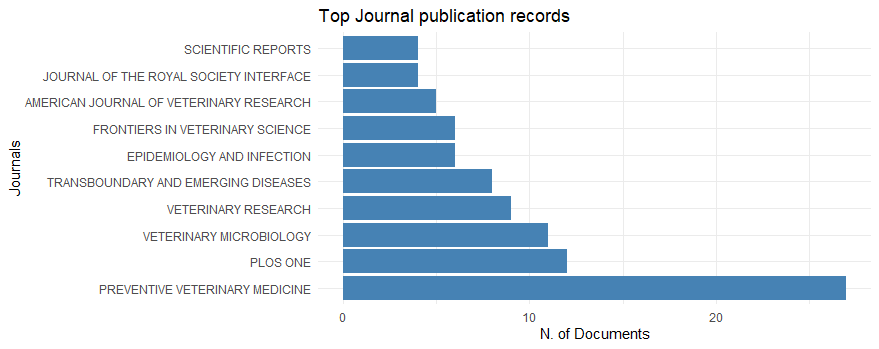

Supplement: Supplementary file 2 — Additional file 2: Supplementary Material 2. Top journal list for modelling studies on infectious diseases dynamics in pigs. [file 40813_2020_160_MOESM2_ESM.tiff]
